# A comparative analysis of gaseous phase hydration properties of two lichenized fungi: *Niebla tigrina* (Follman) Rundel & Bowler from Atacama Desert and *Umbilicaria antarctica* Frey & I. M. Lamb from Robert Island, Southern Shetlands Archipelago, maritime Antarctica

**DOI:** 10.1007/s00792-021-01227-y

**Published:** 2021-05-03

**Authors:** Hubert Harańczyk, K. Strzałka, K. Kubat, A. Andrzejowska, M. Olech, D. Jakubiec, P. Kijak, G. Palfner, Angélica Casanova-Katny

**Affiliations:** 1 M. Smoluchowski Institute of Physics, Jagiellonian University, ul. Łojasiewicza 11, 30-348 Cracow, Poland; 2 Malopolska Centre of Biotechnology, Jagiellonian University, Cracow, Poland; 3 Faculty of Biochemistry, Biophysics and Biotechnology, Jagiellonian University, Cracow, Poland; 4 Institute of Botany, Jagiellonian University, Cracow, Poland; 5 Institute of Biochemistry and Biophysics, Polish Academy of Sciences, Warsaw, Poland; 6 Mycological and Mycorrhizal Laboratory, Concepción University, Concepción, Chile; 7 Plant Ecophysiology Laboratory, Faculty of Natural Resources, Catholic University of Temuco, Rudecindo Ortega, 03694 Temuco, Chile

**Keywords:** Antarctica, Cryptogamic species, Polar tundra, Desiccation, Arid

## Abstract

Gaseous phase hydration properties for thalli of *Niebla tigrina* from Atacama Desert, and for *Umbilicaria antarctica* from Isla Robert, maritime Antarctica, were analyzed using ^1^H-NMR relaxometry, spectroscopy, and sorption isotherm analysis. The molecular dynamics of residual water was monitored to distinguish the sequential binding very tightly, tightly, and loosely bound water fractions. These two species differ in hydration kinetics faster for Desert *N. tigrina* [*A*_1_ = 0.51(4); *t*_1_ = 0.51(5) h, *t*_2_ = 15.0(1.9) h; total 0.7 for *p*/*p*_0_ = 100%], compared to Antarctic *U. antarctica* [*A*_1_ = 0.082(6), *t*_1_ = 2.4(2) h, *t*_2_ = [26.9(2.7)] h, total 0.6 for *p*/*p*_0_ = 100%] from humid polar area. The ^1^H-NMR measurements distinguish signal from tightly bound water, and two signals from loosely bound water, with different chemical shifts higher for *U. antarctica* than for *N. tigrina*. Both lichen species contain different amounts of water-soluble solid fraction. For *U. antarctica,* the saturation concentration of water soluble solid fraction, *c*_s_ = 0.55(9), and the dissolution effect is detected at least up to Δ*m*/*m*_0_ = 0.7, whereas for *N. tigrina* with the similar saturation concentration, *c*_s_ = 053(4), this fraction is detected up to the threshold hydration level equal to Δ*M/m*_0_ = 0.3 only.

## Introduction

For the organisms resistant to the extremal dehydration, as for lichenized fungi (Nash et al. 1990; del Prado and Sancho 2000; Harańczyk et al. 2017), or for some insects (Hinton 1951; Cornette and Kikawada 2011; Cornette et al. 2017), which may transform to cryptobiotic form, water behavior at the initial steps of rehydration is critical for active life recovery. For lichens the dehydration resistance covers not only the normal earth conditions but extends even to the exposition on outer space conditions which beside drastic dehydration include also vacuum and full spectrum of irradiation (de Vera et al. 2003,2004; Meeben et al. 2013a, b; Jänchen et al. 2015; de la Torre et al. 2020). Such a redundancy in biological abilities not often occurs in evolutionary processes; therefore, it draws ones attention on physico-chemical bases of dehydration resistance, not only to the final functional effect. This motivates scientists to study of the residual water behavior in thallus as well as monitoring the optical properties of the thallus (Bartak et al. 2018).

There are not so many experimental methods which may monitor remnants of residual water in extremely dry organism, in vivo. Among them are ^1^H-NMR relaxometry, ^1^H-NMR spectroscopy, and sorption isotherm analysis. They allow the analysis of molecular dynamics of water molecules and differentiation of several fractions of residual water present in a cryptobiotic organism (in dehydrated lichenized fungi) (Harańczyk et al. 2006, 2008, 2009, 2012b), in freeze dried photosynthetic membranes (Harańczyk et al. 2015), or in other extremely dry biological systems like DNA-based conducting polymers (Nizioł et al. 2015).

In thalli of some Antarctic lichen species the ^1^H-NMR experiments reveal an unexpected surplus in mobile proton signal appearing with the increased hydration level during a mild rehydration course performed from gaseous phase. This effect may be caused by two reasons, namely, by a simple dilution of water soluble solid fraction, as it is observed in some plant tissues (Harańczyk et al. 1999) by lichenase-induced lichenin decomposition, which is observed in thalli of lichenized fungi (Harańczyk et al. 2016; Bacior et al. 2017); or for higher plants at initial phases of seed imbibition (Harańczyk et al.^1996^).

However, even the lichens populating the sites on continental Antarctica temporarily experience a relatively high humidity (Sadowsky and Ott 2015), so it is interesting whether this behavior is shared with the species populating dry sites characteristic for Atacama Desert. This implies the question what changes in molecular mechanisms of rehydration may characterize the lichens populating drier habitats or populating sites experiencing more humid periods. The supposed lichenin and isolichenin hydrolysis effectivity observed by these authors at rehydration of Antarctic lichens detected as a dissolution process of water soluble solid fraction extends for hydration levels, Δ*m/m*_0_, up to ca. 0.8 (Haranczyk et al. 2016). The hypotesis was that in lichens populating dry habitats such a process may be not so much effective.

## Materials and methods

**Fig. 1:**
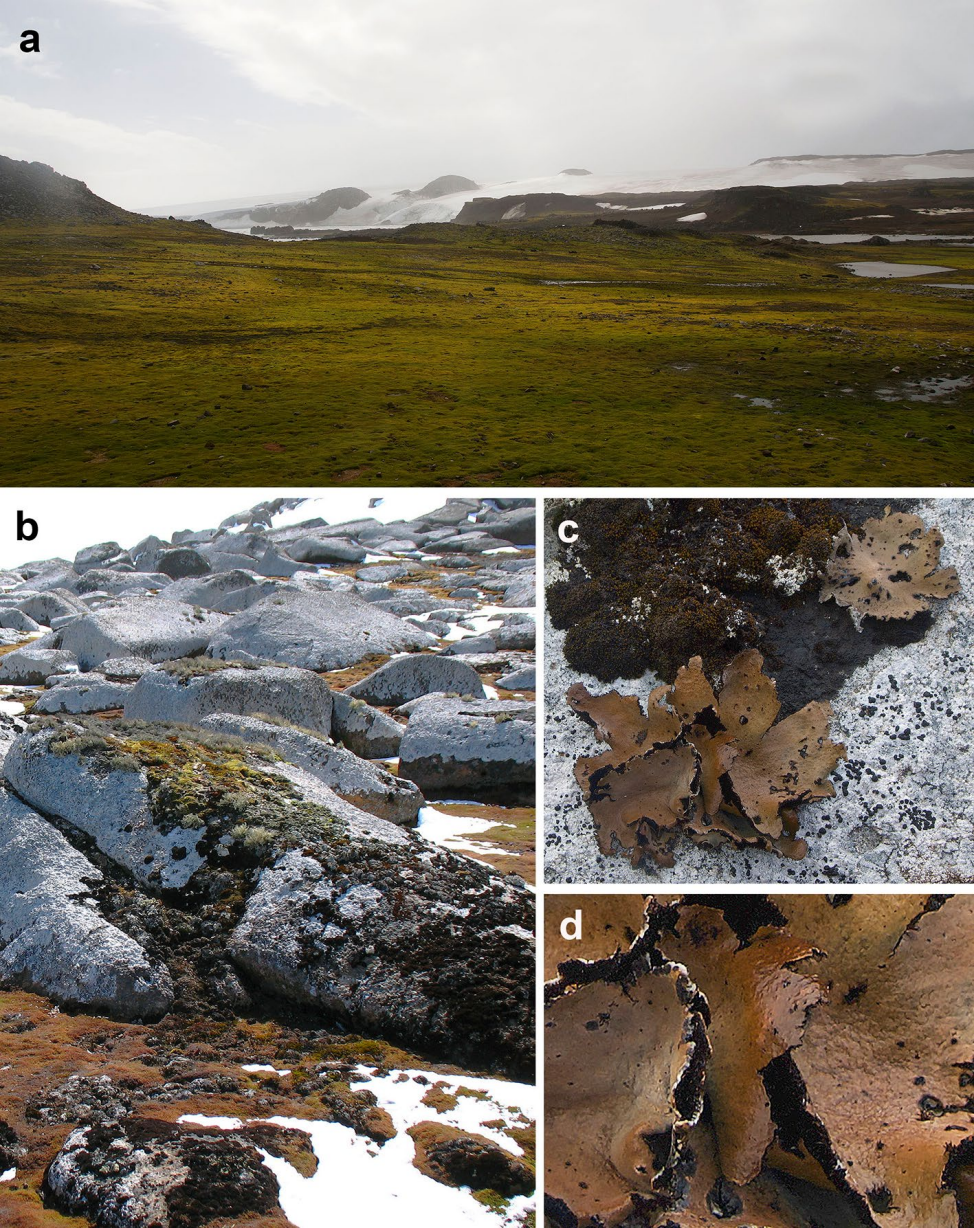
Hydric environment of lichens in Antarctic tundra, Robert Island, South Shetland Archipelago, maritime Antarctica; **a** ice-free area with extensive cryptogam vegetation; **b** colony of saxicolous lichens in snow-melting zone; **c** thalli of *Umbilicaria antarctica* in situ; **d** close up of hydrated thallus of *Umbilicaria antarctica*

The thalli of foliose lichen *Umbilicaria antarctica* (Fig. 1), a chlorolichen formed by an algae of the genus Trebouxia (Romeike et al. 2002) were collected from the site on rocks of Robert Island, South Shetlands Archipelago, maritime Antarctica, on July 7th, 2018, during Chilean 54. ECA (54 Expedición Científica Antártica). Robert Island is the third Island of the South Shetland Island Archipelago on the north part of the west side of the Antarctic Peninsula. The maritime climate is characterized by temperatures between − 2° and 5 °C during the summer season with high photosynthetically active radiation (PAR circa 2000 μmol·m^−2^ s^−1^) (Casanova-Katny et al. 2010). The vegetation is characterized by large stands of cryptogamic species (lichens and bryophytes) and by *Deschampsia antarctica*, the Antarctic grass, is the only one vascular plants colonizing ice free area on the island (Torres-Mellado et al. 2011).

**Fig. 2:**
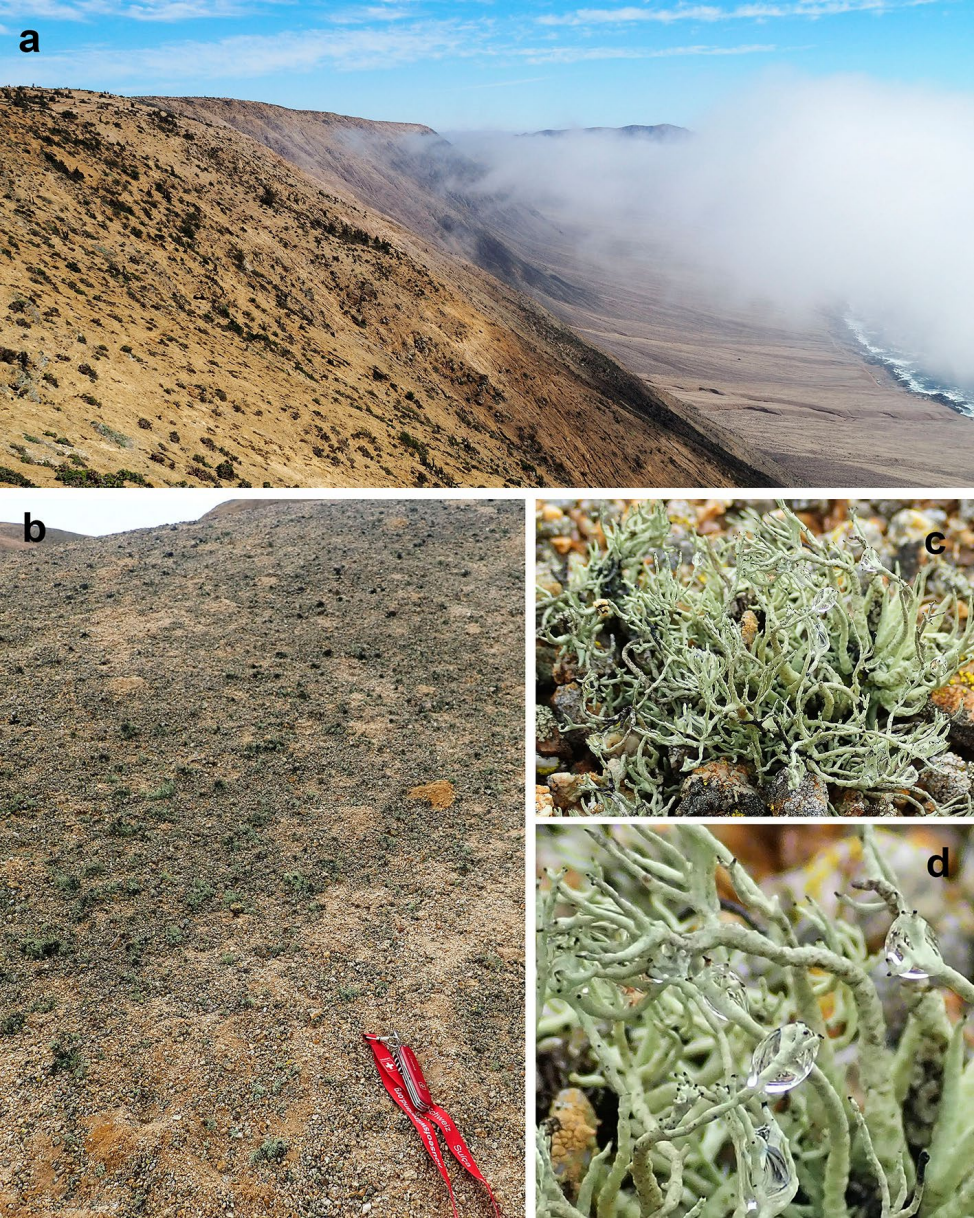
Hydric environment of lichens in coastal desert, Pan de Azúcar National Park, Northern Chile; **a** coastal slope and incoming fog; **b** colony of terricolous lichens in coastal fog zone; **c** individual thallus of *Niebla tigrina* in situ; **d** close up of hydrated thallus of *Niebla tigrina*

The second species, we used for this experiment is the fruticose lichen *Niebla tigrina* (Rundel and Bowler 1978) (Fig. 2), an endemic species collected during the Atacama Desert expedition on November the 7th, 2017, in Las Lomitas area of the Parque Nacional Pan de Azúcar, Chañaral, Atacama Desert, Chile. The lomas formations, correspond to a high topography coastline, with characteristics flora and fauna, largely defined by a distance from coast and elevation with respect to the marine inversion-derived fog layer or to camanchaca (Rundel et al. 1996). Air temperature reaches 13 °C in winter and 20 °C in Summer, with a maximal value of 26 °C; with high relative humidity 80–85%. Few years’ precipitation can exceed 10 mm, but in some “El Niño” years, when sea surface temperature anomalies are positive, extreme precipitation events occur with higher rainfall (Rundel et al. 1996; Thompson et al. 2003). The selection criterion for the lichen species was the sufficient amount of material to be able to carry out the analyzes (0.5–1.0 g), unfortunately we were not able to collect the other foliose species from the Atacama Desert with sufficient mass of material.

Before the hydration courses the thalli were incubated over silica gel (*p*/*p*_0_ = 0%) during 72 h. After the dehydration procedure the hydration courses were performed from the gaseous phase. Humidity was controlled by placing the dry thalli over the surface of the saturated solutions of LiCl (*p*/*p*_0_ = 11%), KC_2_H_3_O_2_ (*p*/*p*_0_ = 23%), CaCl_2_ (*p*/*p*_0_ = 32%), K_2_CO_3_ (*p*/*p*_0_ = 44%), Na_2_Cr_2_O_7_ (*p*/*p*_0_= 52%), NH_4_NO_3_ (*p/p*_0_ = 63%), Na_2_S_2_O_3_ (*p*/*p*_0_ = 76%), Kr_2_CrO_3_ (*p*/*p*_0_ = 88%), Na_2_SO_4_ (*p*/*p*_0_ = 93%), K_2_SO_4_ (*p*/*p*_0_= 97%), and over the water surface (*p*/*p*_0_ = 100%).

After completing the hydration courses, the dry mass, *m*_*0*_, of the sample was determined after heating at 70 °C for 72 h. Relatively low temperature were used for heating, to avoid thermal degradation of thallus constituents (Gaff 1977).

^1^H-NMR free induction decays (FIDs) were recorded using WNS HB-65 high power relaxometer (Waterloo NMR Spectrometers, St. Agatha, Ontario, Canada). The resonance frequency was 30 MHz (at *B*_0_ = 0.7 T); the transmitter power was 400 W; and the pulse length *π*/2 = 1.25 μs. FIDs were acquired and averaged over 2000 accumulations. The hydration time-courses and NMR measurements were performed at room temperature (*t* = 22 °C).

^1^H-NMR spectra were collected on Bruker Avance III 300 spectrometer (Bruker Biospin), operating at the resonance frequency 300 MHz (at *B*_0_ = 7 T), with the transmitter power used equal to 400 W. The pulse length was *π*/2 = 1.5 μs, bandwidths 300 kHz, and repetition time was 2 s. Hydration courses and NMR experiments were performed at room temperature (*t* = 22 °C).

The data obtained were analyzed using the FID analyzing procedure of the two-dimensional (in time domain) NMR signal-analyzing CracSpin program written at the Jagiellonian University, Cracow (Weglarz and Harańczyk 2000), or by commercially available fitting software Origin.

## Results

### Hydration kinetics

**Fig. 3:**
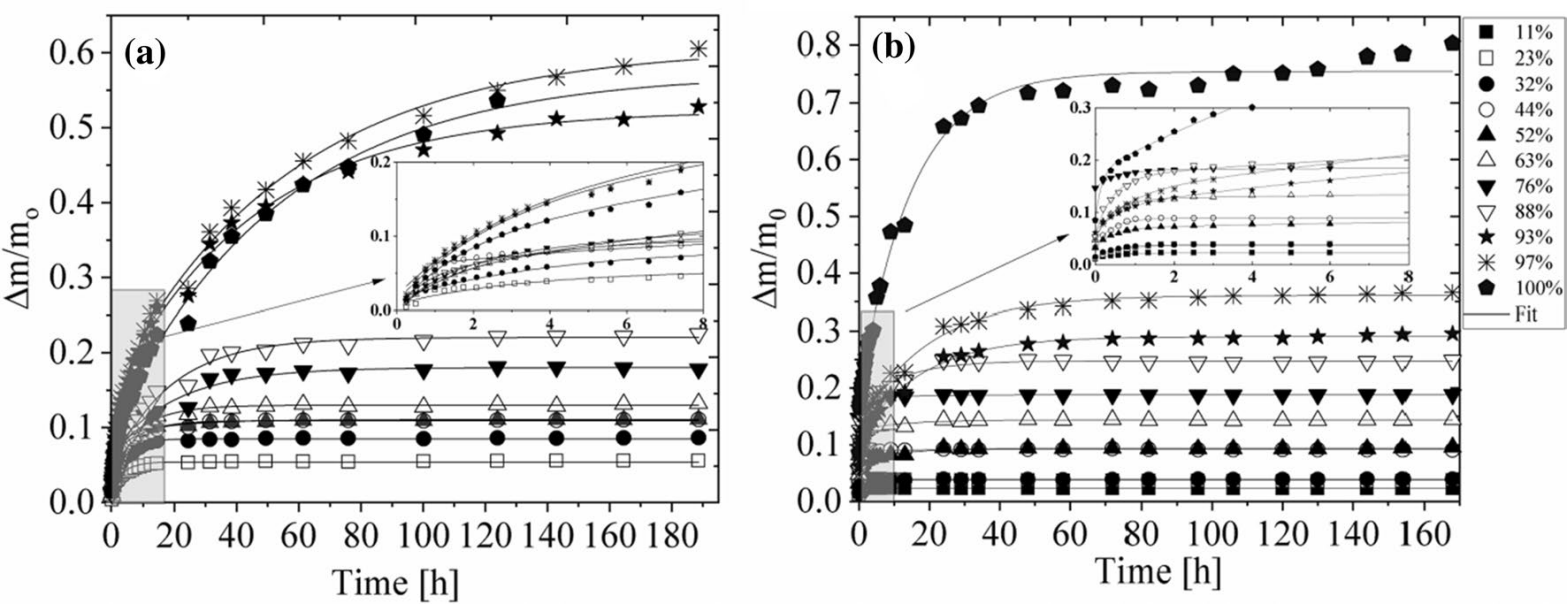
Gaseous phase hydration of the *Umbilicaria antarctica* (**a**), and *Niebla tigrina* (**b**) thalli. The hydration courses were performed at different values of relative humidity *p/p0*, and recorded as relative mass increase expressed in units of dry mass ∆*m/m*_0_. Target humidity: *p/p*_0_ = 11%—closed squares, *p/p*_0_ = 23%—open squares, *p/p*_0_ = 32%—closed circles, *p/p*_0_ = 44%—open circles, *p/p*_0_ = 52%—closed triangles, *p/p*_0_= 63%—open triangles, *p/p*_0_ = 76%—closed reversed triangles, *p/p*_0_ = 88%—open reversed triangles, *p/p*_0_ = 93%—close starlets, *p/p*_0_ = 97%—asterisks, *p/p*_0_ = 100%—closed pentagon. The error bars are within the plot symbols. First 8 h of the course on enlargement

The gaseous phase hydration courses for the air humidity which varied in the range between *p*/*p*_0_ = 11%, and 32%, for *N. tigrina* thallus, and between *p*/*p*_0_ = 23% and 32%, for *U. antarctica*, were fitted well by an one exponential function (see Fig. 3) according to

1a $$ \frac{\Delta m}{{m_{0} }} = A_{0}^{h} + A_{1}^{h} * \left( {1 - {\text{exp}}\left( { - \frac{t}{{t_{1}^{h} }}} \right)} \right), $$

where Δ*m/m*_0_ is the hydration induced relative mass increase expressed in units of dry mass, *m*_0_, $t_{1}^{h}$ is the hydration time, $A_{1}^{h}$ is the saturation hydration level at given humidity, and *A*_*0*_^*h*^ is the hydration level over the silica gel surface (*p*/*p*_0_ = 0%).

For both lichen species investigated, at the relative air humidity equal and higher than *p*/*p*_0_ = 44%, a second, slower hydration component is detected and the hydration courses are fitted well by a superposition of two exponential functions (see Fig. 3) according to

1b $$ \frac{\Delta m}{{m_{0} }} = A_{0}^{h} + A_{1}^{h} * \left( {1 - {\text{exp}}\left( { - \frac{t}{{t_{1}^{h} }}} \right)} \right) + A_{2}^{h} \times \left( {1 - {\text{exp}}\left( { - \frac{t}{{t_{2}^{h} }}} \right)} \right), $$

where $t_{1}^{h}$, $t_{2}^{h}$, are the hydration times, $A_{1}^{h}$, $A_{2}^{h}$, are the saturation hydration levels at given humidity for both hydration components (for tightly and loosely bound water fractions, respectively); and $A_{0}^{h}$ is the hydration level in the atmosphere over silica gel (*p*/*p*_0_ = 0%) corresponding to a very tightly bound water fraction.

Loosely and tightly bound water fractions may be distinguished by their proximity to inner as well as to outer surfaces of the thallus solid matrix, and, thus, by molecular water mobility.

The hydration level over the silica gel surface, *A*_*0*_, is non-zero, and is equal *A*_0_ = 0.051(4) for *N. tigrina*; and for *U. antarctica* is equal to 0.008(5); however, for dehydration courses, the obtained value is higher and is equal *A*_0_ = 0.021(3).

For *U. antarctica* and for *N. tigrina* the gaseous phase hydration courses show sequential binding of a very tightly bound water fraction ($A_{0}^{h}$*)*, a tightly bound water (*A*_*1*_^*h*^), and subsequently a loosely bound water pool $\left( {A_{2}^{h} } \right)$. However, the investigated two lichen species differ in kinetics of gaseous phase hydration.

For *N. tigrina* thalli the hydration time for tightly bound water fraction, equals $t_{1}^{h}$ = 0.51(5) h, whereas for *U. antarctica,* this hydration time is equal $t_{1}^{h}$ = 2.42(29) h. The proportion of tightly bound water fraction, expressed in units of dry mass, *m*_0_, for *N. tigrina* is equal to *A*_1_ = 0.054(4), and for *U. antarctica* is equal *A*_1_ = 0.082(6), which is a value significantly higher.

The loosely bound water component binds with much slower rate, and for *N. tigrina* the hydration time is equal to $t_{2}^{h}$ =15.0(1.9) h, whereas for Antarctic *U. antarctica,* the hydration of loosely bound water fraction is even slower and the rehydration time is equal to $t_{2}^{h}$ =26.9(2.7) h*.*

**Fig. 4:**
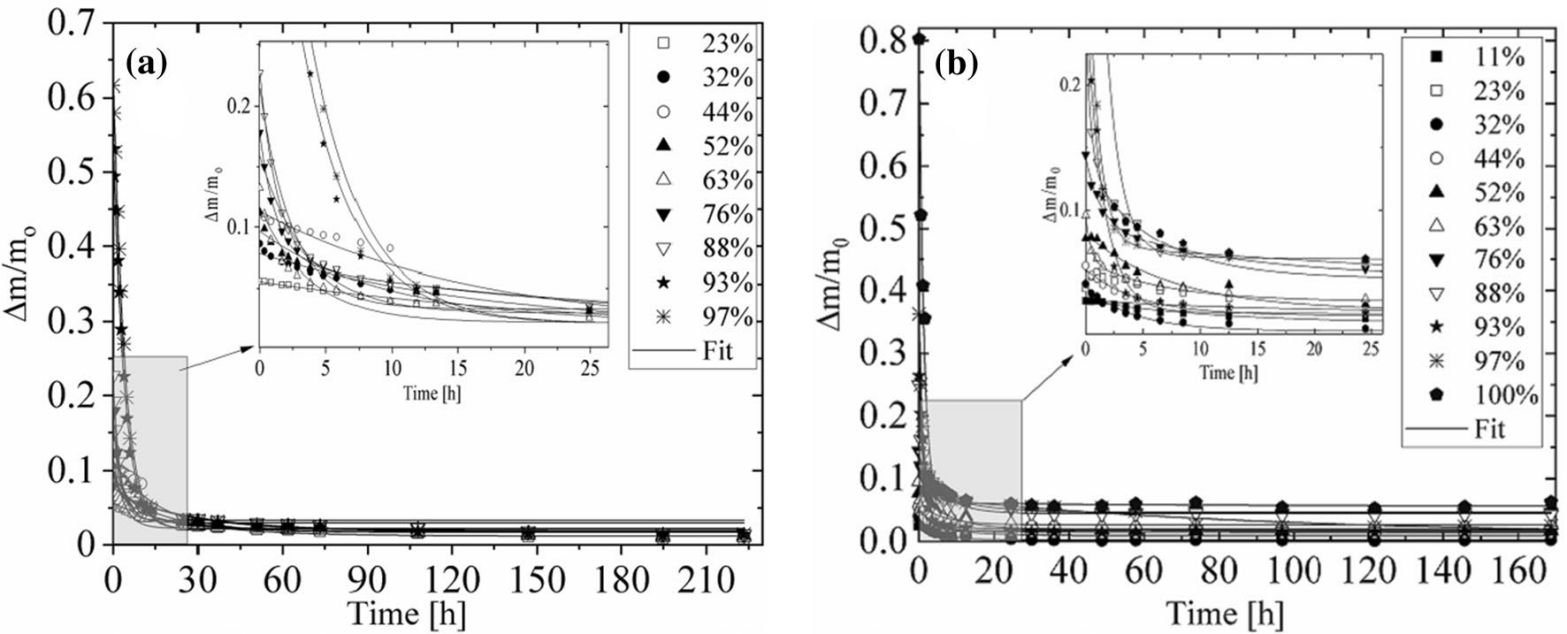
Dehydration to gaseous phase of the *U. antarctica* thalli (**a**) and of the *N. tigrina* thalli (**b**). The dehydration courses were performed for the samples hydrated from gaseous phase at different values of relative humidity *p/p*_0_, and recorded as relative mass increase expressed in units of dry mass ∆*m/m0*. Target humidity: *p/p*_0_ = 11%—closed squares, *p/p*0 = 23%—open squares, *p/p*_0_ = 32%—closed circles, *p/p0* = 44% – open circles, *p/p*_0_ = 52%—closed triangles, *p/p*_0_ = 63%—open triangles, *p/p*_0_ = 76%—closed reversed triangles, *p/p*_0_ = 88%—open reversed triangles, *p/p*_0_ = 93%—close starlets, *p/p*_0_ = 97%—asterisks, *p/p*_0_ = 100%—closed pentagon. The error bars are within the plot symbols. First 26 h of the course on enlargement

In contrast to hydration process, the dehydration course for *N. tigrina* and for *U. antarctica* thallus is well described by a mono exponential function:

1c $$ \frac{\Delta m}{{m_{0} }} = A_{0}^{h} + A_{1}^{h} *\left( {1 - {\text{exp}}\left( { - \frac{t}{{t^{d} }}} \right)} \right), $$

where *A*_0_ = 0.028(2) for *N. tigrina* and *A*_0_ = 0.021(3) for *U. antarctica* is a hydration level remaining in thallus after the dehydration course, the average saturation dehydration level for loosely and for tightly bound water fraction *A*^d^ = 0.164(6) for *N. tigrina*, and *A*^d^ = 0.202(6) for *U. antarctica*, and, *t*^d^ = 5.03(59)*h* for *N. tigrina*, and *t*^d^ = 9.81(1.02) h for *U. antarctica* is a dehydration time (see Fig. 4).

The total hydration level of the lichen sample can be expressed as a sum of all three water fractions:

2 $$ C^{h} = A_{0}^{h} + A_{1}^{h} + A_{2}^{h} . $$

At *p*/*p*_0_ = 100%, the total hydration level, *C*^*h*^, for *N. tigrina* is equal to 0.754, whereas for *U. antarctica* it is lower and is equal to 0.59.

With the increasing air humidity, the gaseous phase hydration courses show a change in behavior for both investigated organisms. The total hydration level increases abruptly for the relative humidity exceeding the certain level, i.e., for Antarctic *U. antarctica* the total hydration level, *C*^*h*^, up to *p/p*_0_ = 88% does not exceed 0.22 and at *p/p*_*0*_ = 93% it increases more than two times reaching *ca*. 0.5 with the maximal value of 0.59 at *p/p*_0_ = 100%.

For the thalli of *N. tigrina* coming from Atacama Desert hydrated at *p/p*_0_ = 100% the total hydration level, *C*^*h*^, increases more than two times as compared with the total hydration level at *p/p*_0_ = 97%, where it is equal to 0.36.

### Sorption isotherm

The total saturation hydration level, *C*^*h*^, expressed as a function of the relative air humidity, *p/p*_0_, (see Eq. 2), was taken for construction of sorption isotherm.

**Fig. 5:**
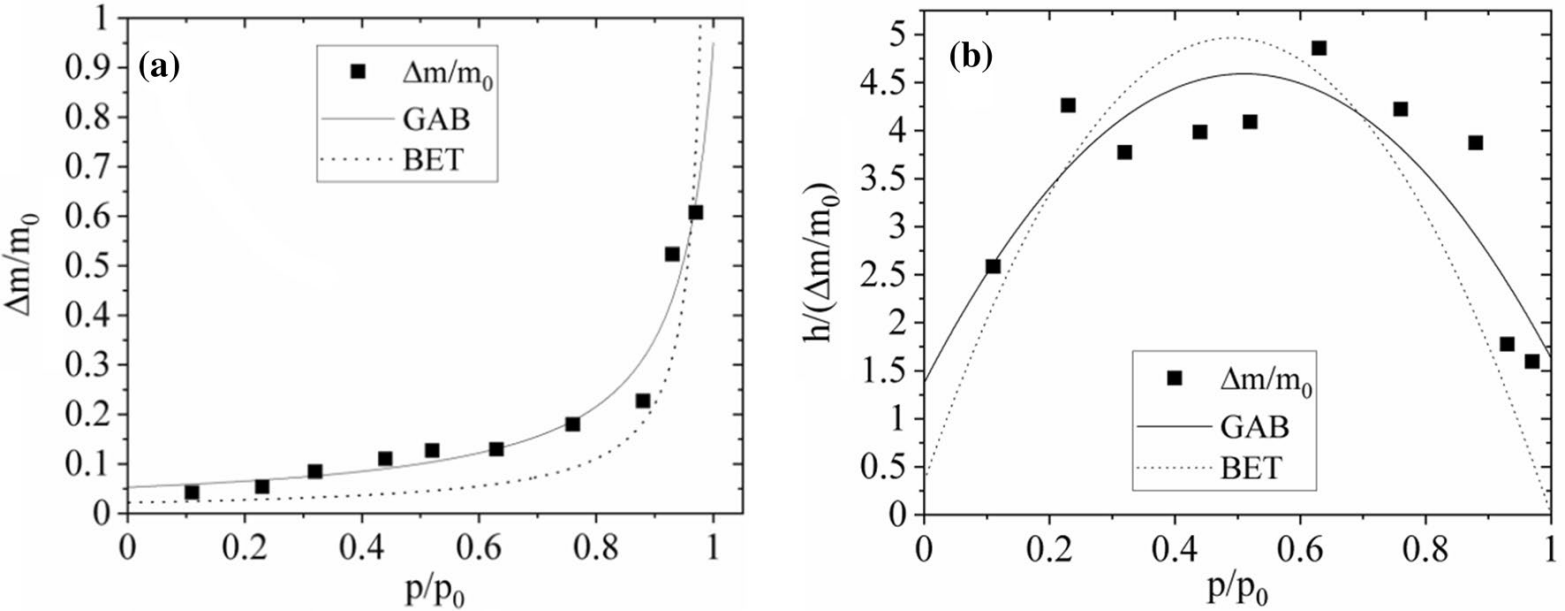
Sorption isotherm (**a**) and parabolic form of GAB model used to fit sorption isotherm (**b**) for the *U. antarctica* thallus (closed squares); dotted line—fitted BET model, solid line—fitted GAB model (Eq. 3). For BET model the sorption isotherm is expressed by $\frac{\Delta m}{{m_{0} }} = \frac{\Delta M}{{m_{0} }} \times \frac{{b_{1} h}}{{\left( {1 - h} \right) \times \left( {1 + b_{1} h - h} \right)}}$

**Fig. 6:**
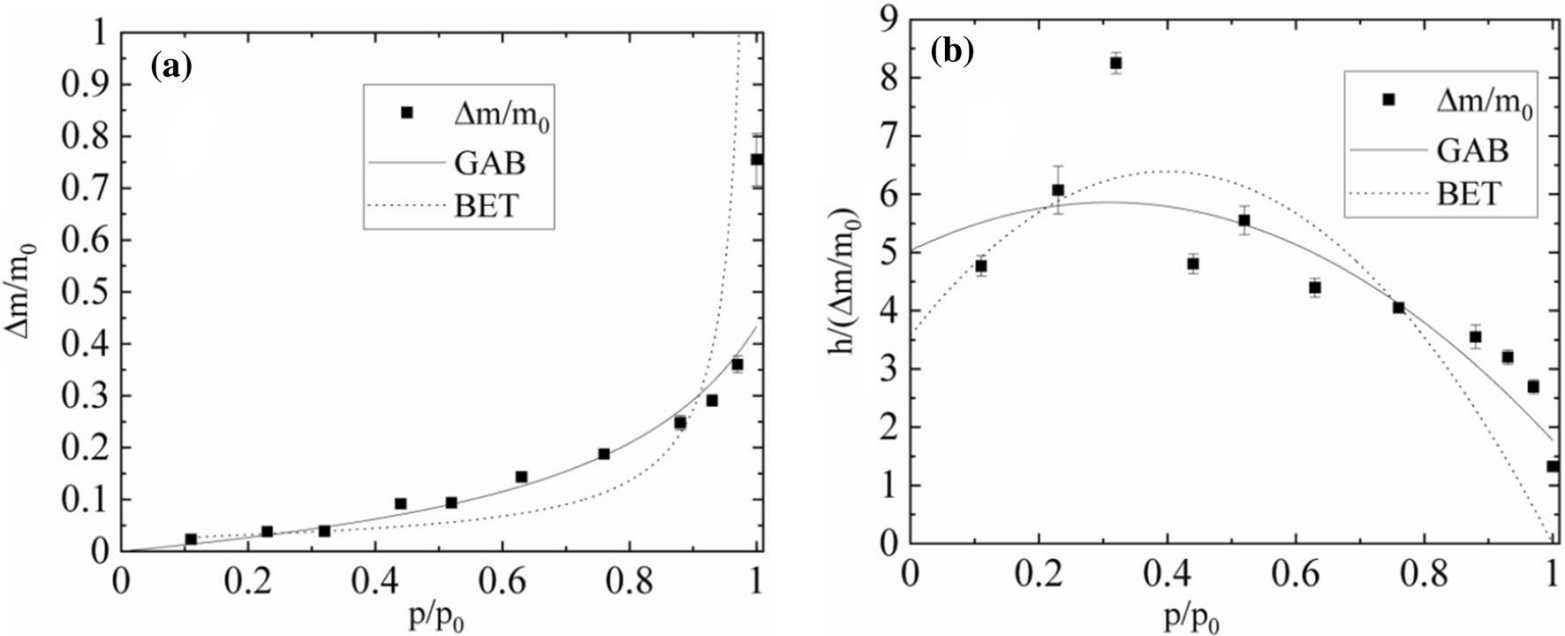
Sorption isotherm (**a**) and parabolic form of GAB model used to fit sorption isotherm (**b**) for the *N. tigrina* thallus (closed squares); dotted line—fitted BET model, solid line—fitted GAB model (Eq. 3). For BET model the sorption isotherm is expressed by $\frac{\Delta m}{{m_{0} }} = \frac{\Delta M}{{m_{0} }} \times \frac{{b_{1} h}}{{\left( {1 - h} \right) \times \left( {1 + b_{1} h - h} \right)}}$

For *U. antarctica* the sorption isotherm is sigmoidal in form (Fig. 5a), but for *N. tigrina* presents only a part of sigmoidal function (Fig. 6a). Such a form of sorption isotherm usually is fitted well by the theoretical multilayer sorption models considering two types of water binding sites. The ‘primary’ water binding sites which are bound directly to the surfaces of the system investigated, i.e., thallus surface in case of lichens, and the ‘secondary’ water binding sites which are bound to the previously bound water molecules, or sometimes to the surface water binding sites with much smaller affinity, as it is in case of more hydrophobic surfaces. The defined by sorption isotherm models water fractions may not be identical with tightly and loosely bound water fractions distinguished by sorption kinetics. The ratio of binding sites covered by *n* water molecules, expressed in units of binding sites covered by *n−*1 water molecules, equals $b = S_{n} /S_{n - 1} |_{h = 1}$. For multilayer sorption processes, two theoretical models are usually considered, namely classic Brunauer–Emmett–Teller (BET) model (Brunauer et al. 1938), and newer Dent (or Guggenheim-Anderson-DeBoer) model. For the Dent (GAB) model (Dent 1977), the fits we obtained the reasonably good quality of the fits, whereas the quality of the fits for BET model was lower.

Sorption isotherm for GAB model is expressed by

3 $$ \frac{\Delta m}{{m_{0} }} = \frac{\Delta M}{{m_{0} }}*\frac{{b_{1} h}}{{\left( {1 - bh} \right)*\left( {1 + b_{1} h - bh} \right)}}, $$

where *h* is relative humidity, *p/p*_0_, expressed in absolute units, Δ*M/m*_0_ is the mass of water saturating primary binding sites. The ratio of, *S*_0_, the number of empty primary binding sites on the surface in units of sites with one water molecule, *S*_1_, at *h* = *1* is expressed by the reciprocal of *b*_1_: $1/b_{1} = S_{0} /S_{1} |_{h = 1}$.

For *N. tigrina* the relative mass of water saturating primary binding sites is equal to Δ*M/m*_0_ = 0.07(1). This value is very close to $A_{0}^{h}$ fitted from hydration kinetics courses. For *U. antarctica *Δ*M/m*_0_ = 0.054(8).

Model parameter *1/b*_1_, is a measure of the number of unoccupied primary water binding sites at *h* = 1, and, thus, the indirect measure of the surface hydrophobicity. For *N. tigrina* this parameter has a relatively high value and is equal *1/b*_1_ = 0.35(4), whereas for *U. antarctica* it is close to zero, 1/*b*_1_ ≈ 0, suggesting that the surfaces of *U. antarctica* are highly hydrophilic.

For *N. tigrina* the model parameter *b* for GAB model, monitoring the tendency of droplet formation at the hydration course is equal *b* = 0.88(12), which is the number close to that for *U. antarctica* for which it is equal *b* = 0.908(29).

To test the relevance of the model applied, the sorption isotherm is usually presented in parabolic form (see Fig. 6):

4 $$\frac{h}{{\Delta m/m_{0} }} = A + Bh - Ch^{2} ,$$

where model parameters Δ*M/m*_0_, b, b_1_ are connected with *A*, *B*, *C* by the formulas

5a $$ b = \frac{{\sqrt {B^{2} + 4AC} - B}}{2A}, $$

5b $$ b_{1} = \frac{B}{A} + 2b, $$

5c $$ \frac{\Delta M}{{m_{0} }} = \frac{1}{{Ab_{1} }}, $$

For BET model parameter *b* = 1 by definition. This means that for hydrating systems in which the multilayer sorption is exactly described by BET model the parabolic form of sorption isotherm is equal zero either for *h* = 0 or for *h* = 1. The surplus in this value over 0 for *h* = 1 is a measure of the applicability of Dent (GAB) model.

For all thalli measured the value of *h*/(Δ*m/m*_*0*_) significantly exceeds zero for *h* = 1, what shows that *N. tigrina* and *U. antarctica* gaseous phase hydration is much better described by Dent than by BET sorption isotherm model.

However, in comparison to the results for *U. antarctica* in case of *N. tigrina* thallus coming from Atacama Desert the applicability of both multilayer sorption models seem to be restricted for the relative humidity level exceeding *p*/*p*_0_ = 97%.

### ^1^H-NMR relaxometry

For *N. tigrina* thallus hydrated up to Δ*m*/*m*_0_ = 0.12 the ^1^H-NMR free induction decay (FID function) is fitted well by a superposition of one Gaussian component coming from restricted in mobility protons of thallus solid matrix, and one exponentially relaxing signal from mobile protons mainly from tightly bound water:

6a $$ {\text{FID}}\left( t \right) = S*{\text{exp}}\left[ { - \left( {\frac{t}{{T_{{{\text{2S}}}}^{*} }}} \right)^{2} } \right] + L_{1} *{\text{exp}}\left( { - \frac{t}{{T_{{{\text{2L1}}}}^{*} }}} \right), $$

where *S* is the amplitude, *T*_2s_^*^ is the 1/e-decay time constant for solid Gaussian component of FID signal; *L*_1_ is the amplitude of mobile proton signal, and *T*_2L1_^*^ the relaxation time for exponential signal component.

**Fig. 7:**
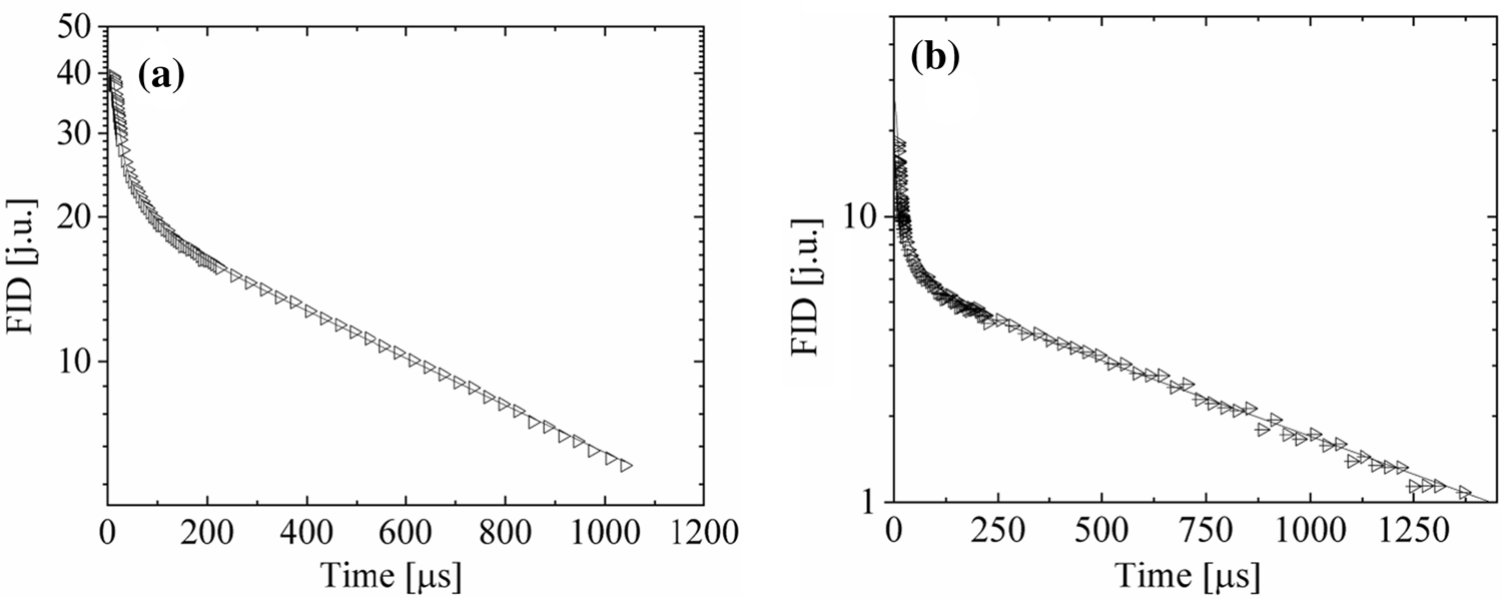
1H-NMR FID function recorded at 30 MHz for the (**a**) *U. antarctica* thallus hydrated to Δ*m/m*_0_ = 0.30, and (**b**) *N. tigrina*, hydrated to the relative mass increase Δ*m/m*0 = 0.18; pulse length π/2 = 1.25 μs; the superposition of Gaussian function and two exponents (Eq. 6b) is fitted (dotted line)

As the hydration level reaches the value Δ*m/m*_0_ = 0.16, the second exponentially relaxing signal of more mobile (loosely bound water) protons arises, and the ^1^H-NMR FID function is expressed by

6b $$ {\text{FID}}\left( t \right) = S*{\text{exp}}\left[ { - \left( {\frac{t}{{T_{{{\text{2S}}}}^{*} }}} \right)^{2} } \right] + L_{1} *{\text{exp}}\left( { - \frac{t}{{T_{{{\text{2L1}}}}^{*} }}} \right) + L_{2} *{\text{exp}}\left( { - \frac{t}{{T_{{{\text{2L2}}}}^{*} }}} \right), $$

where *L*_2_ is the amplitude, and *T*_2L1_^*^ is the effective spin–spin relaxation time for loosely bound water signal. Figure 6 shows the ^1^H-NMR free induction decay (FID) for *U. antarctica* thallus hydrated to Δ*m/m*_0_ = 0.30 (Fig. 7a), and for *N. tigrina* thallus hydrated to Δ*m/m*_0_ = 0.18 (Fig. 7b).

**Fig. 8:**
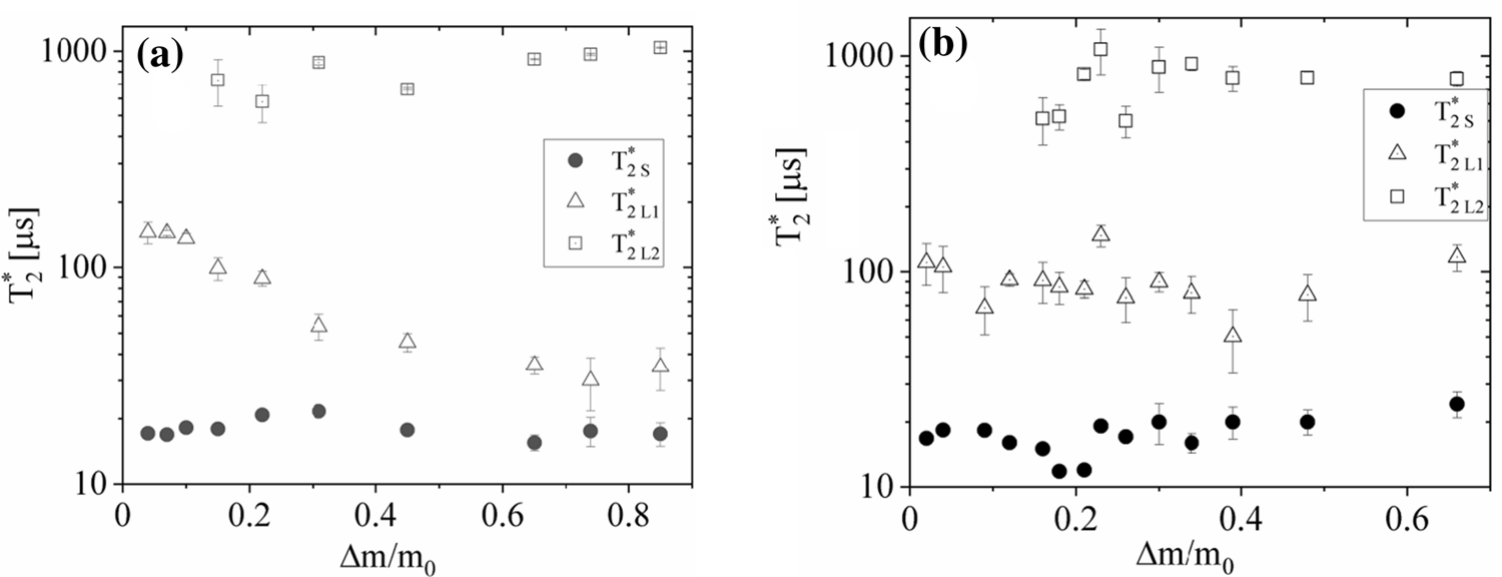
Gaseous phase hydration dependence of proton FID relaxation times for **a** *U. antarctica*, and **b** *N. tigrina* thallus. Solid Gaussian, S, component—closed circles, tightly bound water, *L*1, component—open triangles, and loosely bound water, *L*2, fraction—open squares

With the increased hydration level of thallus the signal coming from immobilized protons of the thallus matrix does not change much, and is fitted well by a Gaussian function (Fig. 8), suggesting that the structure and molecular dynamics of thallus solid matrix is not much altered at the gaseous phase hydration process. ^1^H-NMR FIDs for many Antarctic lichen thalli often reveal a presence of the characteristic “beat pattern” (Weglarz et al. 2000). In such a case much better fit for a rapidly decaying, initial part of FID function is an Abragam function (Abragam 1961), being a product of sinus and Gaussian function which combination in frequency domain represents much more realistic case of rapid decrease to zero in local dipolar magnetic fields for the finite distances (Derbyshire et al. 2004). However, a “beat pattern” was not detected in the presented measurements.

Slowly decaying signal coming from mobile protons is fitted by a superposition of two exponential function indicating that the two fractions of mobile protons may be distinguished. The signal from less mobile proton fraction, *L*_1_, component with *T*_2L1_^*^≈ 100 μs may be connected partially with lipids, and partially with tightly bound water fraction (Harańczyk et al. 2015), and is detected in many other extremely dry biological systems (Harańczyk et al. 1999, 2008, 2009, 2012a; b, c).

The *L*_2_ signal of more mobile proton component coming from water loosely bound on thallus surfaces relaxes with *T*_2L2_^*^≈ 1000 μs, a value significantly shortened by *B*_0_ inhomogeneities, and for still higher hydration level averages also with the free water fraction (Fig. 8).

The shortening effect of *T*_2_ spin–spin relaxation times in FID experiment by *B*_0_ inhomogeneities is given (Timur 1969):

7 $$ \frac{1}{{T_{2}^{*} }} = \frac{1}{{T_{2} }} + \frac{{\gamma \Delta B_{0} }}{2}, $$

where *T*_2_ is spin–spin relaxation time, *γ* is gyromagnetic ratio, and Δ*B*_0_ is a change in magnetic field *B*_*0*_ within the sample.

The two detected mobile proton systems are differentiated by their mobility and, thus, by their binding and/or proximity to the solid thallus surfaces. This means that intracellular water as well as extracellular water fraction usually contributes to both these water fractions.

### The ^1^H-NMR analysis water soluble solid fraction

**Fig. 9:**
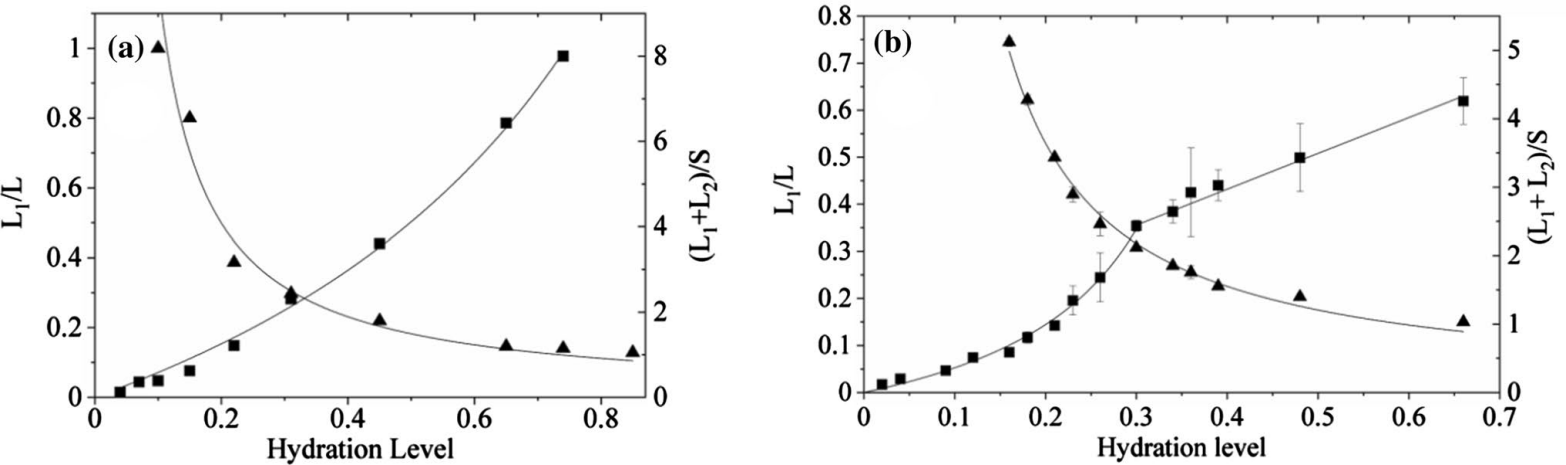
Total mobile proton signal (for tightly bound and loosely bound water) expressed in units of solid, (*L*1 + *L*2*)/S*, for gaseous phase hydration, Δ*m/m*0, dependence (black squares); and the less mobile liquid signal to total liquid signal ratio, *L*1*/L* (black triangles), for **a** *U. antarctica*, and for **b** *N. tigrina* thalli. The solid line are function fitted (see Text)

Figure 9 shows the hydration dependence of ^1^H-NMR signal for *N. tigrina* and *U. antarctica* thallus, expressed as the mass increase in units of the dry mass, Δ*m/m*_0_. The hydration dependence of mobile proton signal amplitude expressed in units of solid signal amplitude, *L/S*, measured for *U. antarctica* thalli in whole range of hydration levels investigated, and for *N. tigrina* thalli for initial part of hydration range is not linear in form (Fig. 9a, b), and is fitted well by a rational function (Harańczyk et al. 2016; Bacior et al. 2017). The surplus in mobile ^1^H-NMR signal component may be caused by a presence of solid water soluble fraction of thallus, which dissolves with the increased hydration level of the sample.

If in the sample there is no liquid fraction trapped in pores of dry matrix, the sample is hydrated by the mass, Δ*m*, of water, and if *m*_cd_ is the mass of dissolved water-soluble solid fraction present in the system, *m*_cd_ = (*c*_s_/1−*c*_s_)Δ*m*, where *c*_s_ is the saturation concentration of the water soluble solid fraction, the intensities of the liquid, *L*, and the solid, *S*, signal components may be written as Harańczyk et al. (1999)

8a $$ L = \alpha_{{{\text{H}}_{{2}} {\text{O}}}} \rho_{{{\text{H}}_{{2}} {\text{O}}}} \Delta m + \alpha_{{{\text{cd}}}} \rho_{{\text{c}}} m_{{{\text{cd}}}} $$

and

8b $$ S = S_{0} - \alpha_{{{\text{cu}}}} \rho_{{\text{c}}} m_{{{\text{cd}}}} , $$

and

8c $$ S_{0} = \alpha_{{\text{s}}} \rho_{{\text{s}}} m_{0} , $$

where $\alpha_{{{\text{H}}_{{2}} {\text{O}}}}$, *α*_cd_, *α*_cu_ are the proportionality coefficients describing the effective contribution of a given proton pool to the total ^1^H-NMR signal for water, for water-soluble solid fraction in liquid, and in solid phase, respectively, *α*_s_ is the responsible proportionality coefficient for solid matrix of thallus; the $\rho_{{{\text{H}}_{{2}} {\text{O}}}}$ and *ρ*_c_, are proton densities for water and for water-soluble solid fraction, respectively, *ρ*_s_ is the averaged proton density for solid matrix of lichen thallus; *m*_0_ is the mass of solid matrix of the thallus, which in case of the absence of water ‘sealed’ in pores is the dry mass of the sample. The slope of the *L/S* hydration dependence measured in case of the absence of water-soluble solid fraction expressed as

9 $$ k = \frac{{\alpha_{{\text{L}}} \rho_{{\text{L}}} }}{{\alpha_{{\text{S}}} \delta \rho_{{\text{S}}} }}. $$

Let’s define the coefficient γ, the water-soluble solid fraction proton density to water proton density ratio: *γ* = (*ρ*_c_/$\rho_{{{\text{H}}_{{2}} {\text{O}}}}$), and *δ* = (*ρ*_s_/$\rho_{{{\text{H}}_{{2}} {\text{O}}}}$) thus, the total signal from liquid component may be expressed in units of solid component, *L/S*, as

10 $$ L/S\left( {\frac{\Delta m}{{m_{0} }}} \right) = \frac{{\left( {k + \frac{{\alpha_{{{\text{cd}}}} }}{{\alpha_{{\text{s}}} }}\frac{{\rho_{{\text{c}}} }}{{\rho_{{\text{s}}} }}\frac{{c_{{\text{s}}} }}{{1 - c_{{\text{s}}} }}} \right)\Delta m/m_{0} }}{{1 - \frac{{\alpha_{{{\text{cu}}}} }}{{\alpha_{{\text{s}}} }}\frac{{\rho_{{\text{c}}} }}{{\rho_{{\text{s}}} }}\frac{{c_{{\text{s}}} }}{{1 - c_{{\text{s}}} }} \times \Delta m/m_{0} }}. $$

Assuming that the decrease of the NMR signal for dissolved water-soluble solid fraction is comparable to that for water, we get (*α*_cd_/*α*_s_) ≈ *k*, which means that water soluble solid fraction if dissolved it is so effective as water, and if the proportionality coefficient for undissolved water-soluble solid fraction is similar to that for solid matrix of thallus, (*α*_cd_/*α*_s_) ≈ 1, we get

11 $$ L/S\left( {\frac{\Delta m}{{m_{0} }}} \right) = \frac{{\left( {k + \frac{\gamma }{\delta }\frac{{c_{{\text{s}}} }}{{1 - c_{{\text{s}}} }}} \right)\Delta m/m_{0} }}{{1 - \frac{\gamma }{\delta }\frac{{c_{{\text{s}}} }}{{1 - c_{{\text{s}}} }} \times \Delta m/m_{0} }}, $$

The values of coefficients *α* may be decreased by the presence of paramagnetic ions in solution or on the surfaces of solid matrix. For the systems free of electron paramagnetic centers *α*_*i*_ = *1* (Witek et al. 2010). However, in many biological systems solid NMR signal may be decreased by the presence of endogenous electron paramagnetic ions, e.g., manganese (Khanna et al. 1983).

Bound water differs in mobility depending on the proximity to the solute surface. In some microheterogeneous systems even tends to arrange in more or less defined layers (Murthy and Worthington 1991).

If in the ^1^H-NMR experiment for the system investigated the signal from tightly bound water, *L*_1_, may be distinguished from loosely bound water fraction *L*_2_, and in the absence of water trapped in pores of dehydrated solid matrix, the liquid signal component may be written as

12 $$ L = L_{1} + L_{2} , $$

and

13 $$ L_{i} = \left( {\alpha_{{{\text{H}}_{{2}} {\text{O}}}}^{i} \rho_{{{\text{H}}_{{2}} {\text{O}}}} + \alpha_{{{\text{cd}}}} \rho_{{\text{c}}} \frac{{c_{{\text{s}}} }}{{1 - c_{{\text{s}}} }}} \right)\Delta m_{i} , $$

where de numerator *i* = 1, 2, and ∆*m*_2_ = ∆*m−*∆*m*_1_.

If the proportionality coefficients for mobile and for immobilized water are equal, $\alpha^{1}_{{{\text{H}}_{{2}} {\text{O}}}}$ = $\alpha^{2}_{{{\text{H}}_{{2}} {\text{O}}}}$, and also for water soluble solid fraction is *α*_cd_ = *α*_cu_, the *L*_1_ to *L* ratio may be written as

14 $$ L_{1} /L = \frac{{m_{1} }}{{m_{1} + \left( {1 + \gamma \frac{{c_{{\text{s}}} }}{{1 - c_{{\text{s}}} }}} \right) \times \Delta m_{2} }}, $$

where *m*_1_ is the mass of water saturating tightly bound water pool.

In majority of living organisms surviving the extreme dehydration the water-soluble solid fraction consists of sugars and/or polyols (Hamada et al. 1994; Harańczyk 2003).

The coefficient *γ* does not vary much over biological carbohydrates with the averaged value equal *γ* = 0.598(7). For polyols such a value is quite similar and equals *γ* = 0.715 ± 0.063. If the type of water-soluble solid fraction is not known, the mean value averaged over sugars and polyols is equal *γ* = 0.657(83) (Harańczyk et al. 2016). This allows one the successful fitting of saturation concentration for water soluble solid fraction in unknown lichen.

The combined fit of the solid-to-liquid hydration dependency (11) and less mobile liquid-to-immobilized liquid hydration dependence (14) yields the relative proton density of solid matrix, the relative proton density and the saturation concentration of water soluble solid fraction for the investigated lichen species.

For *N. tigrina* the saturation concentration of water soluble solid fraction is *c*_s_ = 0.53(4), the coefficient *γ* = 0.60(10) has a value characteristic for sugars, whereas *δ* = 0.31(1), the slope was fitted as *k* = 0.90(9), which is significantly different value as for the linear function fitted for the hydration levels at which the whole portion of water soluble solid fraction is already dissolved in given by 5.23(27*)·*Δ*m/m*_0_ + 0.87(9). The slope of the *L*/*S* hydration dependence calculated for hydration levels exceeding Δ*m/m*_0_ > 0.3 at which the whole water soluble solid fraction is completely dissolved is equal 5.24(27).

For *U. antarctica* the saturation concentration for solid water soluble fraction *c*_s_ = 0.55(9), proton density of water soluble solid fraction expressed in units of water proton density *γ* = 0.50(5), proton density of solid matrix expressed in units of water proton density is *δ* = 0.88(12), the presumed slope of *L*/*S* hydration dependence in the absence of water soluble solid fraction is equal to *k* = 3.39(34).

### ^1^H-NMR spectroscopy

For the *N. tigrina* thalli hydrated up to Δ*m/m*_0_ = 0.15 and for the *U. antarctica* thalli hydrated up to Δ*m/m*_0_ = 0.42 the recorded ^1^H-NMR spectrum is a superposition of the broad line component coming from the partially immobilized protons of thallus solid matrix, which may be successfully fitted by Gaussian function, and the narrow line component coming from mobile protons mainly of water bound in thallus, fitted well by one Lorentzian function (Eq. 15a):

15a $$ A\left( \nu \right) = \frac{{A_{{\text{G}}} }}{{\Delta \nu_{{\text{G}}} \times \sqrt {ln4 \times \pi /2} }}{\text{exp}}\left[ { - 2ln4 \times \left( {\frac{{\nu - \nu_{{\text{G}}} }}{{\Delta \nu_{{\text{G}}} }}} \right)^{2} } \right] + \frac{{2A_{{\text{L}}} }}{\pi }\left[ {\frac{{\Delta \nu_{{\text{L}}} }}{{4 \times \left( {\nu - \nu_{{\text{L}}} } \right)^{2} + \Delta \nu_{{\text{L}}}^{{2}} }}} \right], $$

where ∆*ν*_G_ and ∆*ν*_L_ are the half-widths of the NMR line; *ν*_G_ and *ν*_L_ are peak positions; and *A*_*G*_ and *A*_*L*_ are the amplitudes of Gaussian and Lorentzian peaks, respectively.

For the *N. tigrina* thalli hydrated to Δ*m/m*_0_ > 0.15, and *U. antarctica* thalli hydrated to Δ*m/m*_0_ > 0.42 a second Lorenzian in form line component is detected, and a spectrum is fitted well by a superposition of one Gaussian function and two Lorentzian functions differing in peak positions and in line half-widths (Eq. 15b):

15b $$ A\left( \nu \right) = \frac{{A_{{\text{G}}} }}{{\Delta \nu_{{\text{G}}} \times \sqrt {{\text{ln}}4 \times \pi /2} }}{\text{exp}}\left[ { - 2{\text{ln}}4 \times \left( {\frac{{\nu - \nu_{{\text{G}}} }}{{\Delta \nu_{{\text{G}}} }}} \right)^{2} } \right] + \frac{{2A_{{{\text{L1}}}} }}{\pi }\left[ {\frac{{\Delta \nu_{{{\text{L1}}}} }}{{4 \times \left( {\nu - \nu_{{{\text{L1}}}} } \right)^{2} + \Delta \nu_{{{\text{L}}1}}^{2} }}} \right] + \frac{{2A_{{{\text{L2}}}} }}{\pi }\left[ {\frac{{\Delta \nu_{{{\text{L2}}}} }}{{4 \times \left( {\nu - \nu_{{{\text{L2}}}} } \right)^{2} + \Delta \nu_{{{\text{L2}}}}^{{2}} }}} \right] $$

where ∆*ν*_L1_ and ∆*ν*_L2_ are the half-widths of two Lorentzian NMR lines; *ν*_L1_ and *ν*_L2_ are their peak positions; and finally *A*_L1_ and *A*_*L2*_ are the areas under two Lorentzian peaks, respectively.

**Fig. 10:**
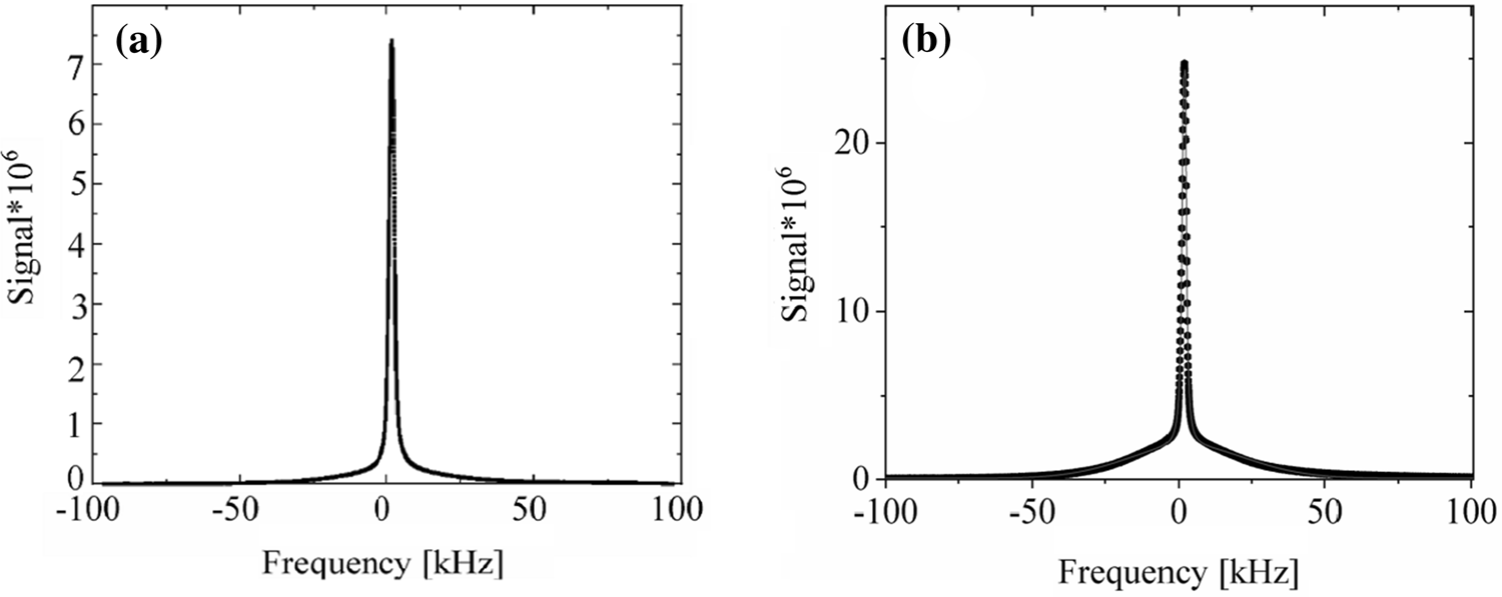
1H-NMR spectrum for **a** *U. antarctica* and **b** *N. tigrina* thalli hydrated to **a** Δ*m/m*_0_ = 0.30 and **b** Δ*m/m*_0_ = 0.18, recorded at 300 MHz—solid squares, Eq. 8b fitted—solid line

**Fig. 11:**
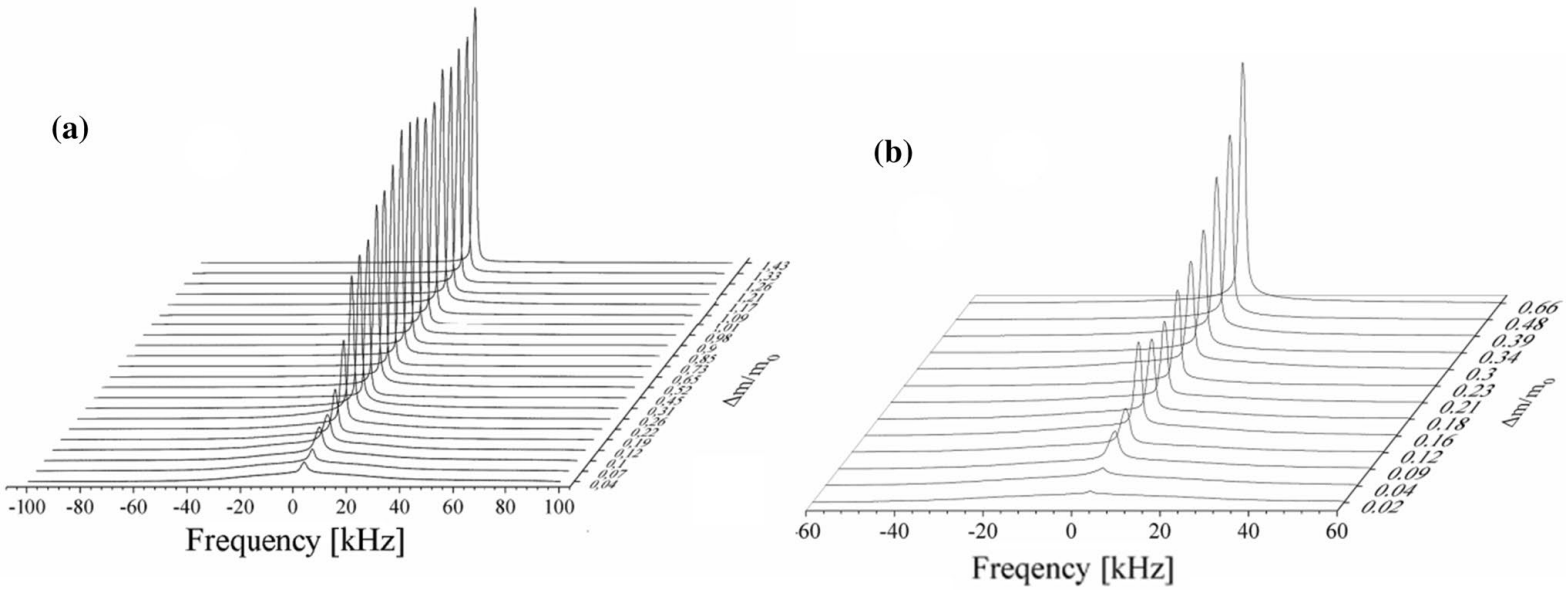
Stacked plot of 1H-NMR spectra for **a** *U. antarctica* and for **b** *N. tigrina* thalli recorded as a function of hydration level increased, since Δ*m/m*_0_ = 0.02 up to 0.66 for *N. tigrina* and up to 1.43 for *U. antarctica*

Figure 10 shows ^1^H-NMR spectrum for *U. antarctica* thallus hydrated to Δ*m/m*_0_ = 0.30, and for *N. tigrina* thallus hydrated to Δ*m/m*_0_ = 0.18. (Eq. 15b), whereas the stacked plots of the ^1^H-NMR spectra measured as a function of hydration level for *U. antarctica* and for *N. tigrina*, samples are presented in Fig. 11.

As no “beat pattern” in FID function was detected, also no “hat-like” (Derbyshire et al. 2004) function was fitted, and the fits of Gaussian function for the partially immobilized protons of thallus solid matrix were sufficiently effective (Abragam 1961). Table 1a, b shows the spectral parameters for ^1^H-NMR spectra recorded as a function of increased hydration level. Either for *N. tigrina* or for *U. antarctica* the half-width of the solid Gaussian line component is equal to ∆*ν*_G_ ≈ 45 kHz, and does not change significantly with the increasing hydration level, up to Δ*m/m*_0_ ≈ 0.7 for *N. tigrina*, and up to Δ*m/m*_0_ ≈ 1.4 for *U. antarctica*.

**Table 1:** Parameters fitted to the ^1^H-NMR spectra (a) for *Niebla tigrina*, and (b) for *Umbilicaria antárctica* thalli recorded at room temperature as a function of hydration level

*m/m*_0_	$\nu G\;\left[ {{\text{Hz}}} \right]$	$\Delta \nu G\;\left[ {{\text{kHz}}} \right]$	$\nu_{{L_{1} }} \left[ {{\text{Hz}}} \right]$	$\Delta \nu_{{{\text{L}}_{{1}} }} \left[ {{\text{kHz}}} \right]$	$\nu_{{{\text{L}}_{{2}} }} \left[ {{\text{Hz}}} \right]$	$\Delta \nu_{{{\text{L}}_{{2}} }} \left[ {{\text{kHz}}} \right]$	*A*L1*/S*	*A*L2*/S*	*A*L*/S*
(a) *Niebla tigrina*									
0.02	3570 (35)	57.66 (11)	2594 (33)	5.05 (12)			0.05 (1)		0.05 (1)
0.04	3153 (32)	56.48 (10)	2231 (14)	4.75 (5)			0.10 (1)		0.10 (1)
0.09	4576 (40)	54.78 (11)	2096 (12)	2.86 (2)	2758 (12)	1.00 (7)	0.20 (1)	0.02 (1)	0.24 (1)
0.12	1336 (30)	53.28 (8)	1546 (13)	2.12 (1)	2357 (10)	1.29 (3)	0.22 (1)	0.07 (1)	0.27 (1)
0.16	3202 (39)	51.49 (11)	1495 (6)	1.40 (1)	2195 (4)	1.04 (1)	0.24 (1)	0.15 (1)	0.39 (1)
0.18	2357 (43)	51.38 (12)	1489 (6)	1.49 (1)	2214 (4)	1.00 (1)	0.28 (1)	0.15 (1)	0.43 (1)
0.21	3042 (46)	49.31 (12)	1502 (4)	1.32 (1)	2169 (3)	0.93 (1)	0.33 (1)	0.18 (1)	0.51 (1)
0.23	2994 (52)	48.60 (14)	1523 (5)	1.39 (1)	2221 (3)	0.91 (1)	0.39 (1)	0.19 (1)	0.58 (1)
0.3	3306 (71)	43.27(19)	1513 (4)	1.39 (1)	2205 (3)	0.84 (1)	0.70 (1)	0.28 (1)	0.98 (1)
0.34	2534 (79)	40.97 (21)	1571 (3)	1.30 (1)	2202 (2)	0.76 (1)	0.98 (1)	0.32 (1)	1.30 (1)
0.39	2334 (80)	41.86 (22)	1539 (3)	1.40 (1)	2201 (2)	0.79 (1)	1.02 (1)	0.33 (1)	1.36 (1)
0.48	3424 (147)	46.52 (39)	1557 (3)	1.25 (1)	2161 (2)	0.75 (1)	1.71 (1)	0.63 (1)	2.34 (2)
0.66	4047 (199)	50.93 (53)	1546 (3)	1.33 (1)	2195 (2)	0.76 (1)	1.80 (1)	0.70 (1)	2.50 (2)
(b) *Umbilicaria antarctica*									
0.02	5854 (72)	48.95 (29)	2517 (3)	4.93 (1)			0.09		0.09
0.04	5082 (27)	53.04 (8)	2654 (6)	2.86 (2)			0.11		0.11
0.07	2980 (16)	50.51 (5)	2575 (3)	2.61 (1)			0.11		0.11
0.10	3486 (21)	50.38 (7)	2114 (2)	3.34 (1)			0.33		0.33
0.12	1263(20)	49.27 (6)	2041 (2)	3.18 (1)			0.37		0.37
0.19	2097 (26)	48.48 (8)	1928 (1)	2.30 (1)			0.56		0.56
0.22	3720 (66)	48.53 (20)	1925 (1)	1.98 (1)			1.02		1.02
0.26	2940 (83)	48.40 (25)	1931 (1)	1.86 (1)			1.51		1.51
0.31	5833 (187)	54.27 (57)	1807 (1)	1.80 (1)			2.51		2.51
0.45	8501 (167)	56.84 (51)	1251 (3)	1.42 (1)	2170 (2)	1.33 (1)	1.11	2.28	3.39
0.52	11593 (534)	45.90 (100)	1412 (3)	1.37 (1)	2220 (2)	1.02 (1)	2.73	2.78	5.51
0.65	5320 (853)	45.27 (98)	1145 (3)	1.21 (1)	2071 (2)	1.13 (1)	2.33	3.41	5.74
0.73	3021 (182)	53.99 (81)	1359 (2)	1.10 (1)	2136 (2)	0.97 (1)	3.61	3.54	7.15
0.82	2998(163)	54.00 (90)	1418 (3)	1.21 (1)	2222 (2)	0.84 (1)	4.75	4.36	9.11

**Fig. 12:**
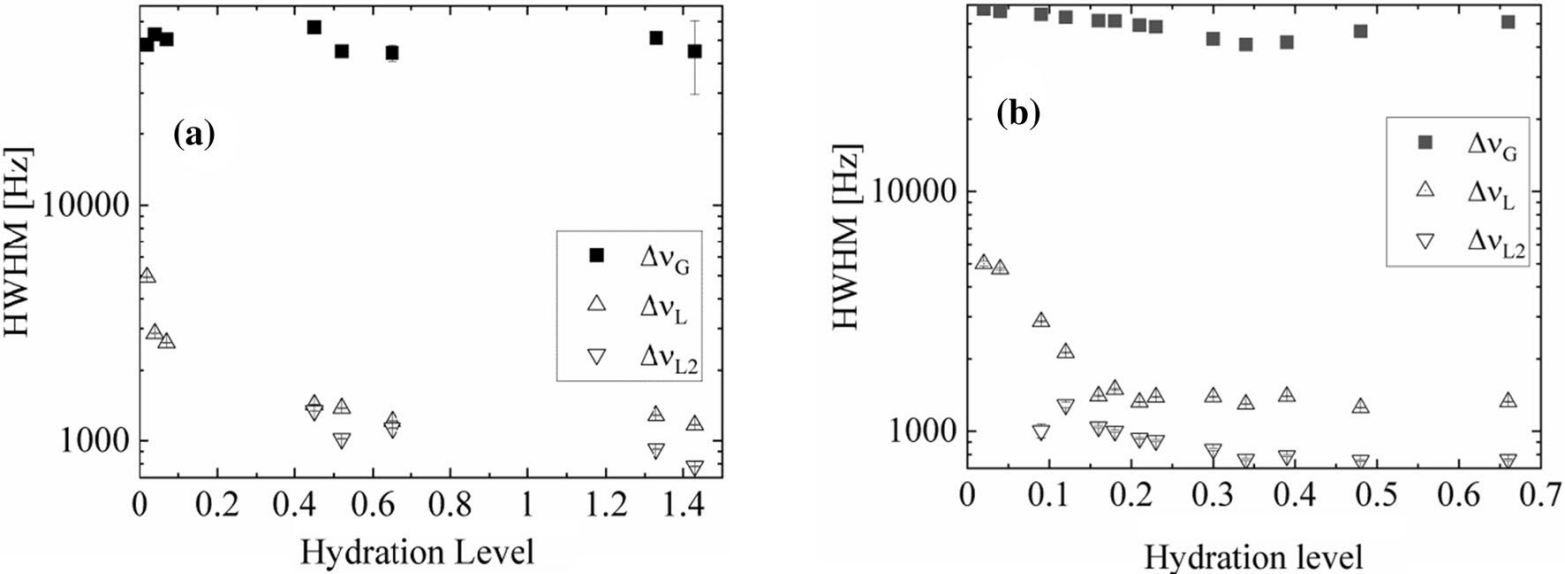
^1^H-NMR line half-widths for **a** *U. antarctica, *and **b** *N. tigrina* thalli expressed as a function of hydration level Δ*m/m*_0_

For *N. tigrina* the half-width (Fig. 12b) of the Lorentzian line, *L*_1_, coming from restricted in mobility tightly bound water fraction, with the increased hydration level of the thallus, decreases, since ∆*ν*_L1_ ≅ 5.05(12) kHz at Δ*m/m*_*0*_ = 0.02, down to ∆*ν*_L1_ ≅ 1.4 at Δ*m/m*_0_ = 0.16 and does not change much for the higher levels of hydration, with the half-width ∆*ν*_L1_ ≅ 1.25(1) kHz at Δ*m/m*_0_ = 0.48. For *U. antarctica* the similar decrease is observed (Fig. 12a).

The half-width of the Lorentzian line, *L*_*1*_, from tightly bound water decreases, since ∆*ν*_L1_ ≅ 4.93(1) kHz at Δ*m/m*_0_ = 0.02 decreases down to ∆*ν*_L1_ ≅ 1.42(1) kHz at Δ*m/m*_0_ = 0.45, and does not change much with the higher hydration level, with the half-width equal ∆*ν*_L1_ ≅ 1.21(1) at Δ*m/m*_*0*_ = 0.82.

The half-width of the Lorentzian *L*_2_ narrower line coming from loosely bound water fraction, with the increased hydration level of the thallus, decreases for *N. tigrina,* since ∆*ν*_L2_ ≅ 1.00(7) kHz at Δ*m/m*_0_ = 0.09 down to ∆*ν*_L2_ ≅ 0.76(1) kHz at Δ*m/m*_0_ = 0.66, whereas for *U. antarctica* it decreases, since ∆*ν*_L2_ ≅ 1.33(1) kHz at Δ*m/m*_0_ = 0.45 down to ∆*ν*_L2_ ≅ 0.84(1) kHz at Δ*m/m*_0_ = 0.82.

The ^1^H-NMR spectrum of *N. tigrina* and *U. antarctica* shows that the *L*_1_ is not a one water fraction, but seems to be an average of tightly and loosely bound water fraction being in fast exchange regime, as its half-width continuously decreases with the increased hydration level.

For higher hydration levels another loosely bound water fraction, *L*_2_, is distinguished with different peak position than that for bound water fraction *L*_1_.

**Fig. 13:**
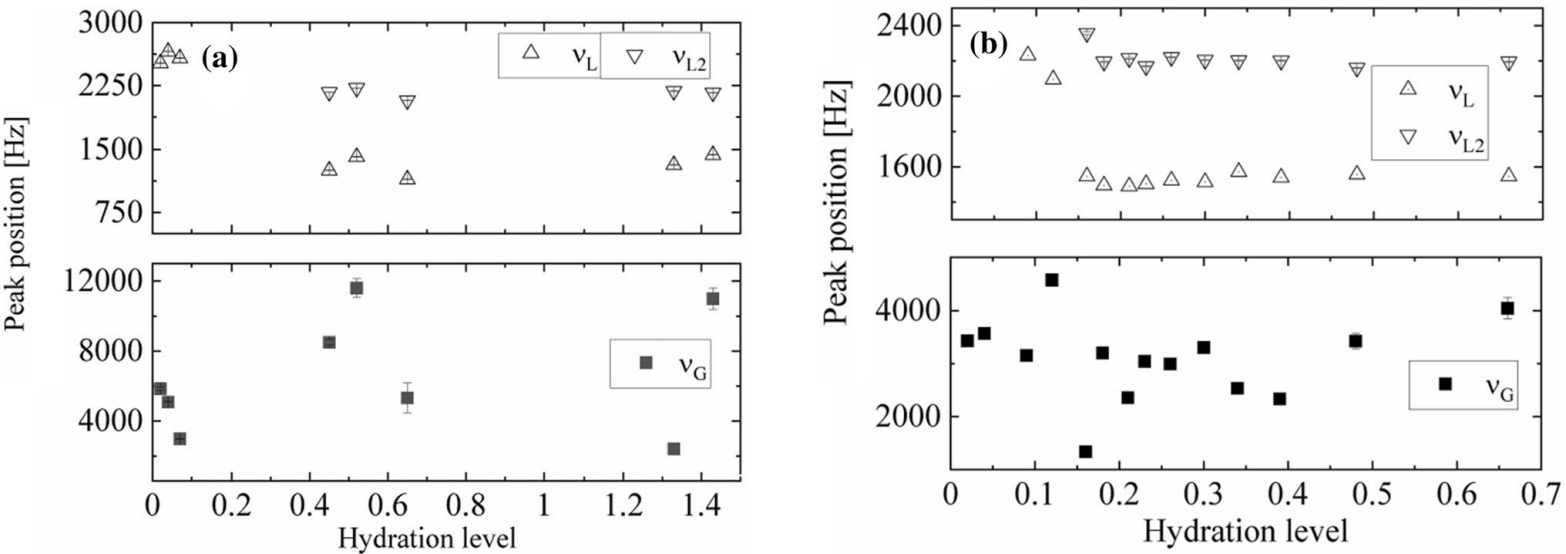
Peak positions of ^1^H-NMR spectra for **a** *U. antarctica* and **b** *N. tigrina* thalli expressed as a function of hydration level Δ*m/m*_0_. Upper plot: two Lorentzian peaks (stable at least up to Δ*m/m0* = 1.43 for *U. antarctica*, and up to 0.7 for *N. tigrina*); lower plot Gaussian peak position

For tightly bound and loosely bound water the peak positions (Fig. 13) of the Lorentzian lines are different, which may be attributed to the difference in chemical shifts. For *N. tigrina* the difference in peak position between *L*_1_ and *L*_2_ line, is equal *ν*_L1_−*ν*_L2_ ≅ 600 Hz, whereas for *U. antarctica* the difference is higher, and is equal *ν*_L1_−*ν*_L2_ ≅ 800 Hz.

**Fig. 14:**
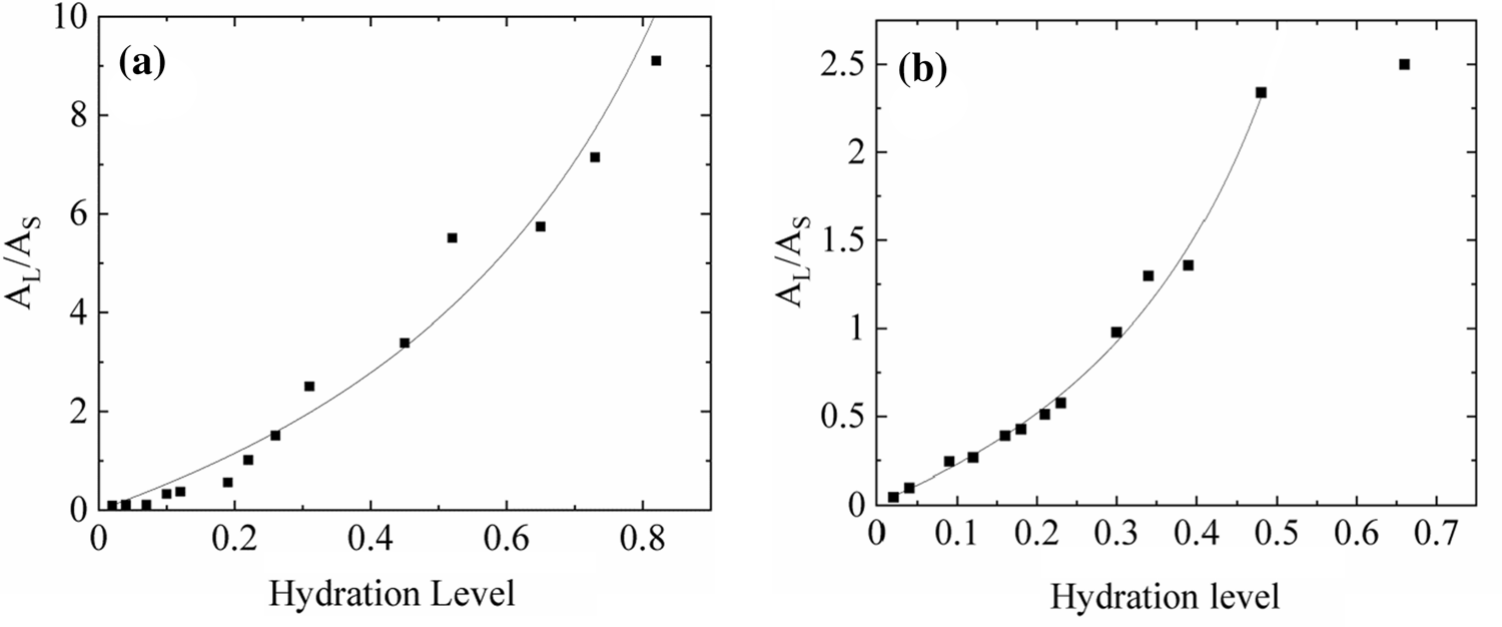
Area under ^1^H-NMR mobile proton line expressed in units of immobilized proton signal area, *A*L/*A*S, for **a** *Umbilicaria antarctica*, and for **b** *N. tigrina* thalli expressed as a function of hydration level Δ*m/m*_0_. The solid line is a rational function fitted (See text)

The total signal coming from mobile protons (tightly and loosely bound water fractions) expressed in units of immobilized proton signal, *L/S*, gradually increases with the increase of the sample hydration level, but the increase is fitted by a rational function as it is for the ^1^H-NMR signal recorded in time domain (Fig. 14).

## Discussion

Antarctic *U. antarctica* and Desert *N. tigrina* thalli significantly differ in gaseous phase hydration rates, although the measured *N. tigrina* specimen comes from the area in which air humidity is relatively high exceeding capillary condensation point (water droplets were sometimes observed on plant surfaces). The hydration rate of tightly bound water fraction for *U. antarctica* is much slower than that for *N. tigrina* as the hydration time, the reciprocal of hydration rate, is equal *t*_1_^h^ = 2.42(29) h versus *t*_1_^h^ = 0.51(5) h, respectively. The tightly bound and loosely bound water fractions differentiated by their molecular mobility are characterized by a different proximity to the inner thallus structures; however, the tendency in hydration rates is similar for both fractions. The hydration rate for loosely bound water fraction is slower for *U. antarctica* than for *N. tigrina*, with the hydration times *t*_*2*_^*h*^ = 26.9(2.7) h versus *t*_2_^h^ = 15.0(1.9) h, respectively.

Sadowsky and Ott (2015) indirectly analyzed gaseous phase hydration kinetics for isolated *U. antarctica* photobiont detecting the dehydration/hydration induced the decay/recovery of maximum quantum yield of PS II chlorophyll a fluorescence. They found that 20-min dehydration to gaseous phase (over silica gel) caused the decrease down to near-zero-level, whereas only 5-min rehydration from gaseous phase (at *p/p*_0_ = 100%) restored this process (Sadowsky and Ott 2012). The low values of hydration times for isolated *Trebouxia* sp. cells recorded by them might be expected for the limited size of photobiont as compared to those detected for extended in shape *U. antarctica* foliose thallus. However, for microbial matt of the foliose green alga *Prasiola crispa* thallus, a free living photobiont of *Turgidosculum complicatulum*, the direct measurement of gaseous phase hydration course showed hydration time much longer and equal to *t*_1_^h^ = 0.37(14) h, with the still much longer hydration times for slower second component hydrating with the hydration time *t*_2_^h^ = 42.6(3.2) h, (Bacior et al. 2017) which is a value rather observed for lichens.

For the cultured photobionts (*Trebouxia* sp.) rather the excess light energy tolerance than the desiccation stress slows down the rehydration process (Determeyer-Wiedmann et al. 2018), suggesting that much longer hydration times for lichen thallus compared to that for algae, either photobionts, or free living species, may be explained rather in terms of extended thallus structure of Antarctic lichen mycobiont, as the volume occupied by cell walls and gelatinous substances which may constitute between 50 and 70% of volume for thalli of 12 species belonging to the lichen family *Umbilicariaceae* populating the sites in Spanish Sistema Central (Valladares et al. 1998).

Presumably the lower hydration rate for two bound water fraction distinguished may be not only connected with the lichen thallus structure, but also with the habitat of the specimens. Hydration time of tightly bound water fraction in *U. antarctica* resembles those for *Ramalina terebrata* with *t*_1_^h^ = 1.24(24) h, (Harańczyk et al. 2012b) for *Leptogium puberulum* from King George Island, with *t*_1_^h^ = 1.6(3) h (Hamada et al. 1994), and for *Turgidosculum complicatulum* with *t*_1_^h^ = 1.45(21) h (Bacior et al. 2017). Among the species from Antarctica only *Cetraria aculeata* from Penguin Island with the hydration time of tightly bound water equal to *t*_1_^h ^= 0.43(10) h (Harańczyk et al. 2016) resembles that for *N. tigrina* from Atacama Desert.

For *U. antarctica* thallus hydrated from gaseous phase up to Δ*m/m*_0_ = 0.75 the presence of water-soluble solid fraction is suggested in whole range of hydration levels. The saturation concentration for solid water soluble fraction is equal *c*_s_ = 0.55(9). This value resembles those for some sugars (as sucrose, galactose, or xylose). For Antarctic *Turgidosculum complicatulum* the saturation concentration of water soluble solid fraction, *c*_s_ = 67(46)%, from NMR relaxometry, and *c*_s_ = 60(13)% from spectroscopy (Bacior et al. 2017); for *Cetraria aculeata* the saturation concentration is equal to *c*_s_ = 57.3(12.0)%, which is the value close to that for averaged carbohydrate and polyol saturation concentration calculated. The observation of these sugars suggests that glucose is transformed to other sugars in lichen thallus (Harańczyk et al. 2016). As in case of *T. complicatulum* the mechanism responsible for the solid matrix dissolution may be the enzymatic degradation process of lichenin (Bacior et al. 2017).

Gaseous phase hydration process for Atacama Desert lichen *N. tigrina* is in pronounced contrast to that observed in case Antarctic lichen species. Although the saturation concentration of water soluble solid fraction is equal *c*_s_ = 0.53(4), which is the value similar to that detected for *U. antarctica*, the hydration dependence of NMR mobile proton signal expressed in units of solid signal shows the pronounced threshold at Δ*m/m*_0_ = 0.3. Above the threshold hydration level (Δ*m/m*_0_ = 0.3) the function describing the ^1^H-NMR signal hydration dependence is no longer described by a rational function but by a simply linear function as it is in a vast majority of hydrated systems.

For hydration levels Δ*m/m*_0_ < 0.3 the hydration dependence is characteristic for the presence of water-soluble solid fraction (is described by a rational function). However, for the hydration levels Δ*m/m*_0_ > 0.3 the hydration is described by a linear function showing the limited contribution of water-soluble solid fraction. The lower contribution of water soluble solid fraction in Atacama Desert *N. tigrina* may have functional reasons, which will be a subject for further research.

## Conclusion

- The gaseous phase hydration process is faster for *N. tigrina*, a Desert species, than that for Antarctic *U. antarctica* from more humid polar area, reflecting water availability in environment. This was found for tightly bound water fraction and for loosely bound water fraction [*A*_1_ = 0.51(4); *t*_1_ = 0.51(5) h, *t*_2_ = 15.0(1.9) h], compared to [*A*_1_ = 0.082(6), *t*_1_ = 2.4(2) h, *t*_*2*_ = (26.9(2.7) h].
- In contrast to gaseous phase hydration the gaseous phase dehydration is described by one averaged process with the dehydration time for *N. tigrina* equal to, *t*^d^ = 5.03(59) h, and for *U. antarctica* equal to *t*^d^ = 9.81(1.02) h.
- Different value of saturation hydration level for tightly bound water fraction for *N. tigrina* and for *U. antarctica*. For *U. antarctica* the mass of water saturating primary water binding sites, Δ*M/m*_0_ = 0.054(8), whereas for *N. tigrina*, it is equal 0.07 ± 0.01 which shows the decreased hydrophilicity of thallus surface for a Desert lichen species.
- Different value of total hydration saturation level. At *p/p*_0_ = 100%, the total hydration level, *C*^*h*^, for *N. tigrina* is equal 0.754, whereas for *U. antarctica,* it is lower and is equal to 0.59.
- Different relative humidity at which the drastic (more than two times) increase in total hydration level, *C*^*h*^, takes place (93% for *U. antarctica*, and 100% for *N. tigrina*).
- The water soluble solid fraction for Antarctic *U. antarctica* with saturation concentration *c*_s_ = 0.55(9) still is detected for the hydration levels at least up to Δ*m/m*_0_ = 0.7, whereas for the *N. tigrina* thallus with the similar saturation concentration *c*_s_ = 0.53(4), water-soluble solid fraction is detected up to the threshold hydration level Δ*M/m*_0_ = 0.3 only.

## Abbreviations

^1^H-NMR: Proton nuclear magnetic resonance.
DNA: Deoxyribonucleic acid
FIDs: Free induction decays
BET: Brunauer–Emmett–Teller model
GAB: Guggenheim-Anderson-DeBoer
